# Quantitative prediction of selectivity between the A_1_ and A_2A_ adenosine receptors

**DOI:** 10.1186/s13321-020-00438-3

**Published:** 2020-05-13

**Authors:** Lindsey Burggraaff, Herman W. T. van Vlijmen, Adriaan P. IJzerman, Gerard J. P. van Westen

**Affiliations:** 1 Division of Drug Discovery & Safety, Leiden Academic Centre for Drug Research, Leiden University, Einsteinweg 55, 2333 CC Leiden, The Netherlands; 2 Janssen Research & Development, Turnhoutseweg 30, Beerse, Belgium

**Keywords:** Selectivity, Selectivity window, Modeling, QSAR, A_1_ adenosine receptor, A_2A_ adenosine receptor, GPCR

## Abstract

The development of drugs is often hampered due to off-target interactions leading to adverse effects. Therefore, computational methods to assess the selectivity of ligands are of high interest. Currently, selectivity is often deduced from bioactivity predictions of a ligand for multiple targets (individual machine learning models). Here we show that modeling selectivity directly, by using the affinity difference between two drug targets as output value, leads to more accurate selectivity predictions. We test multiple approaches on a dataset consisting of ligands for the A_1_ and A_2A_ adenosine receptors (among others classification, regression, and we define different selectivity classes). Finally, we present a regression model that predicts selectivity between these two drug targets by directly training on the difference in bioactivity, modeling the selectivity-window. The quality of this model was good as shown by the performances for fivefold cross-validation: ROC A_1_AR-selective 0.88 ± 0.04 and ROC A_2A_AR-selective 0.80 ± 0.07. To increase the accuracy of this selectivity model even further, inactive compounds were identified and removed prior to selectivity prediction by a combination of statistical models and structure-based docking. As a result, selectivity between the A_1_ and A_2A_ adenosine receptors was predicted effectively using the selectivity-window model. The approach presented here can be readily applied to other selectivity cases.
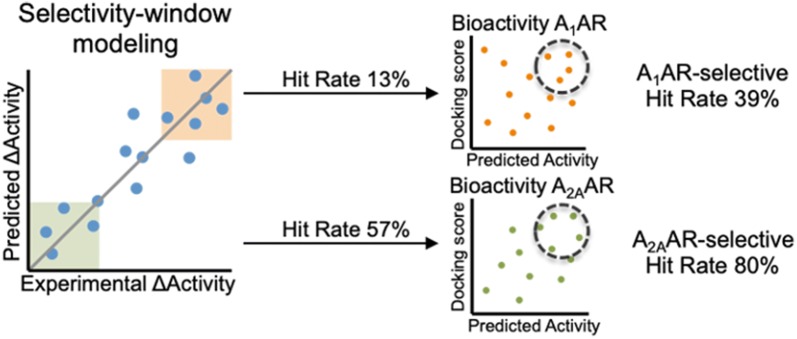

## Introduction

Computational modeling of small molecules in drug discovery is typically focused on modeling their binding affinity or bioactivity. These models can be used to identify active compounds in silico, or to rationalize which chemical groups are correlated with bioactivity. Quantitative structure–activity relationship (QSAR) models can be applied to model compound activity for single proteins, whereas proteochemometrics (PCM) can be applied to model activity for multiple proteins in one single model [1, 2]. Next to statistical models (e.g. machine learning), structure-based models are used to predict and rationalize compound activity. Methods such as docking, molecular dynamics, and free-energy perturbation (FEP) are widely applied to study binding and bioactivity [3–5].

Although modeling of activity is essential in drug discovery, these models often do not take target selectivity into account. The ability to control promiscuity of potential drug candidates is crucial as off-target effects can be avoided this way. Whereas selective drugs are designed to be non-promiscuous, polypharmacological drugs are designed to interact with multiple targets [6]. The development of both polypharmacological and selective drugs requires predictions for more than one target. Polypharmacology and selectivity can both be modeled by machine learning or structure-based approaches that predict the affinity of compounds on multiple targets separately. The resulting bioactivity profile can subsequently be applied to match the desired on-target(s) and to avoid off-target effects. However, this indirect way of predicting selectivity based on predicted bioactivities requires multiple model predictions to calculate one feature: the activity difference of a compound for one target over the other.

Here we explore selectivity modeling for the adenosine receptors, which are members of the class A G protein-coupled receptors (GPCRs). The adenosine receptor family, existing of subtypes A_1_, A_2A_, A_2B_, and A_3_, is involved in many physiological processes including cardiac control and inflammation [7]. The A_1_ and A_2A_ adenosine receptors (A_1_AR and A_2A_AR) both control cyclic adenosine-5′-monophosphate (cAMP) levels in the cell. Activation of A_1_AR results in decreased cAMP levels, whereas A_2A_AR activation increases cAMP levels [8]. These contrary effects justify a need for computational models that can predict selectivity between these two subtypes. A novel method to model selectivity is presented in this study: namely to train machine learning models directly on the differences between experimentally determined affinities.

For the A_2A_AR many crystal structures are available in the Protein Data Bank [9]. More recently, protein crystal and cryo-EM structures for the A_1_AR have been obtained also [10–12]. The availability of structures for both proteins allows for a structure-based comparison of the subtypes to gain insights into selectivity. Previous studies revealed differences between the protein structures that correspond with ligand selectivity of specific chemical structures [10, 11, 13]. For example, the A_2A_AR-selectivity of reference antagonist ZM241385 could be explained by a combination of three structural differences between the A_1_AR and A_2A_AR: a salt bridge at the binding pocket entry, a hydrophobic pocket in the A_1_AR, and a (de)stabilized water network [13]. Furthermore, the A_1_AR-selectivity of xanthine-based antagonists with a bulky substituent has shown to be caused by steric hindrance in the A_2A_AR by residue Met270^7.35^ (Thr270^7.35^ in A_1_AR) [11].

In addition to the availability of crystal structures, many small molecule ligands have been experimentally tested for their activity on one or multiple adenosine receptors. This data has already been exploited to train bioactivity models using classification or continuous statistical models [14, 15]. However, direct modeling of selectivity in the adenosine receptors using statistical models has not yet been reported. Statistical models to predict selectivity are faster than predicting selectivity using time-consuming FEP approaches [5]. However, structure-based methods can give additional mechanistic information on binding and selectivity of compounds, and are in principle not dependent on available bioactivity data of ligands.

In this research a combined approach of QSAR modeling and structure-based docking is presented to model bioactivity for the A_1_AR and A_2A_AR. Moreover, the selectivity between the A_1_AR and A_2A_AR is predicted directly by training on affinity differences (selectivity-window). This is in contrast to methods that were reported up till now that deduced selectivity from predicted bioactivities of separate models. Furthermore, by training a continuous model (regression), the degree of selectivity was calculated in addition to a selectivity class with predefined thresholds. Finally, to enhance the performance of the selectivity models, statistical bioactivity models and structure-based docking were used to exclude inactive compounds.

Our study shows that continuous QSAR models can effectively predict selectivity between the A_1_AR and A_2A_AR. A model trained directly on the difference in affinity between the two proteins, the selectivity-window model, outperformed models that are generally used to predict selectivity: models that are trained on separate bioactivities for the A_1_AR and A_2A_AR.

## Results

### Datasets

**Fig. 1:**
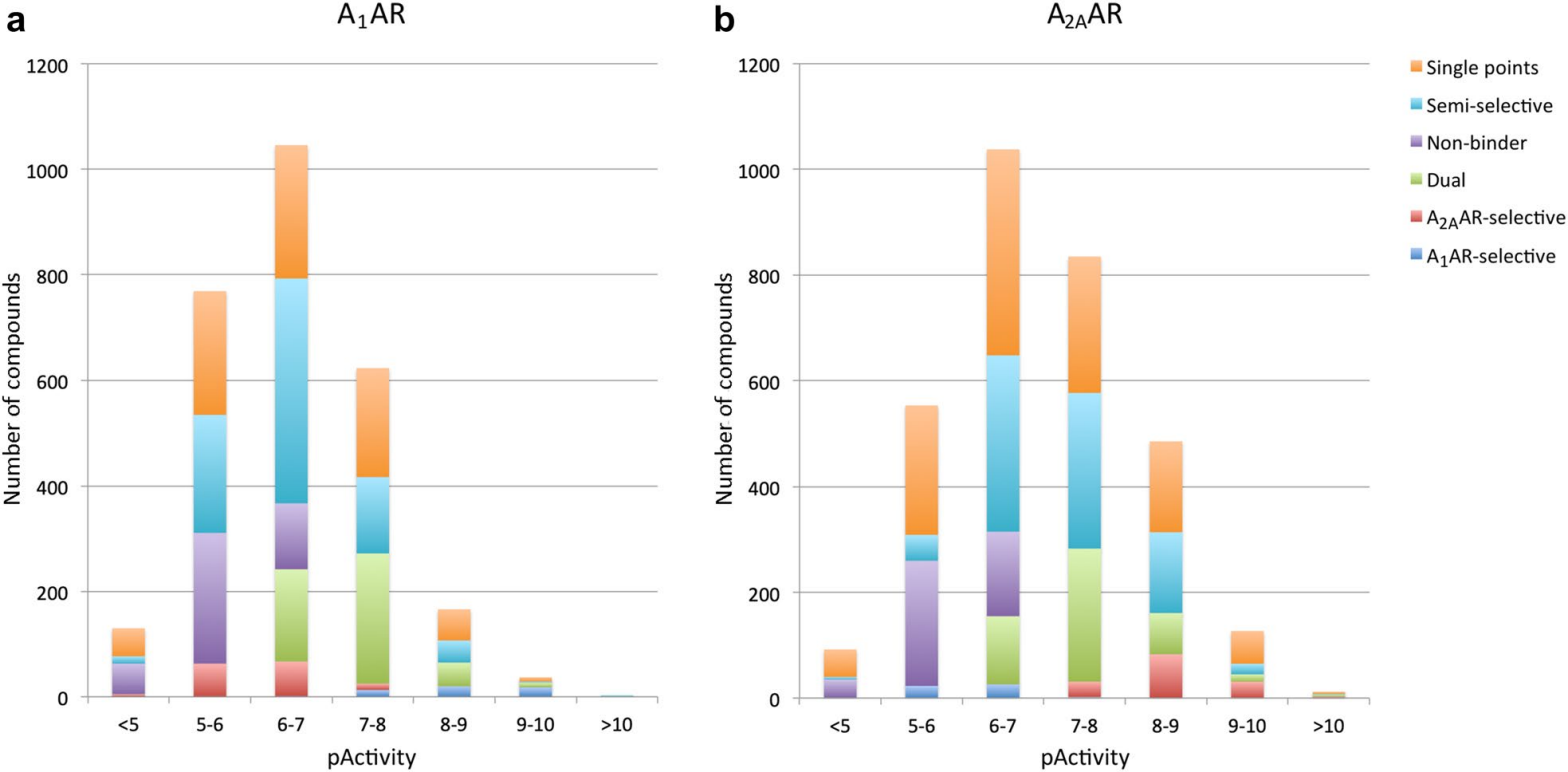
Distribution of activities of the different compound classes for the A_1_AR (**a**) and A_2A_AR (**b**). The compounds from the A_1_AR and A_2A_AR bioactivity datasets that did not belong to any of the classes of the A_1_AR/A_2A_AR dataset are called “single points”

Information on compound-protein interactions (e.g. binding affinity and efficacy) was collected for the human A_1_AR and A_2A_AR. Public bioactivity data was extracted from ChEMBL version 23 [16] and supplemented with in-house data (see Additional file 1). As compounds with a ribose scaffold are often associated with agonistic activity and dicyanopyridines with partial agonism, these compounds were removed to generate an antagonist-focused dataset [17, 18]. Bioactivity values were standardized to pActivity (conceptually similar to the pChEMBL value, an ensemble from pK_i_/IC_50_/EC_50_/K_d_ values [19]), while simultaneously combining data from different labs and assays. The data was subsequently used to compile the following compound datasets: an ‘A_1_AR bioactivity dataset’ (containing bioactivities of compounds on the A_1_AR), an ‘A_2A_AR bioactivity dataset’ (containing bioactivities of compounds on the A_2A_AR) and an ‘A_1_AR/A_2A_AR dataset’ (bioactivities of compounds tested on both the A_1_AR and A_2A_AR). The latter dataset included information on the selectivity of compounds. Compounds were termed ‘selective’ when the difference in activity between the two proteins was more than 100-fold (e.g., A_2A_AR-selective when pActivity A_1_AR = 6.5 and pActivity A_2A_AR ≥ 8.5). The A_1_AR/A_2A_AR dataset consisted of five classes: non-binder (pActivity A_1_AR and A_2A_AR < 6.5), A_1_AR-selective (pActivity A_1_AR ≥ 6.5 and selectivity ≥ 100-fold), A_2A_AR-selective (pActivity A_2A_AR ≥ 6.5 and selectivity ≥ 100-fold), and dual binder (pActivity A_1_AR and A_2A_AR ≥ 6.5 and selectivity ≤ tenfold). Additionally, compounds that had measured bioactivities for both the A_1_AR and A_2A_AR, but did not fit into any of the classes of the A_1_AR/A_2A_AR dataset, were termed ‘semi-selective’ compounds. Compounds that only had measured bioactivity for one receptor and were not present in the A_1_AR/A_2A_AR dataset, but were included in either the A_1_AR bioactivity dataset or A_2A_AR bioactivity dataset were termed ‘single points’. The distribution of activities in the different datasets was comparable (Table 1) and normally distributed in both the A_1_AR and A_2A_AR (Fig. 1). It should be noted that the total number of A_1_AR-selective compounds is about three times smaller than the number of A_2A_AR-selective compounds (50 and 146 compounds, respectively).

**Table 1:** Dataset characteristics: number of compounds, distribution of activities and chemical similarity within the dataset

Dataset	Description	Total number of compounds	Activity (pActivity)				Similarity (tanimoto FCFP4)
			Protein	Min	Median	Max	Mean
A_1_AR bioactivity dataset	Compounds with measured activity for the A_1_AR	2774	A_1_AR	4.05	6.43	10.52	0.18
A_2A_AR bioactivity dataset	Compounds with measured activity for the A_2A_AR	3123	A_2A_AR	4.00	6.91	11.00	0.18
A_1_AR/A_2A_AR dataset	Compounds with measured activity for both the A_1_AR and A_2A_AR with classification A_1_AR/A_2A_AR/dual/non-binder	1106	A_1_AR	4.33	6.52	10.52	0.19
			A_2A_AR	4.30	6.83	10.80	
Semi-selective compounds	Compounds with measured activity for both the A_1_AR and A_2A_AR that do not fit into a class	855	A_1_AR	4.37	6.37	10.02	0.20
			A_2A_AR	4.34	7.09	10.38

### Modeling A_1_AR/A_2A_AR subtype selectivity using classification QSAR models

**Fig. 2:**
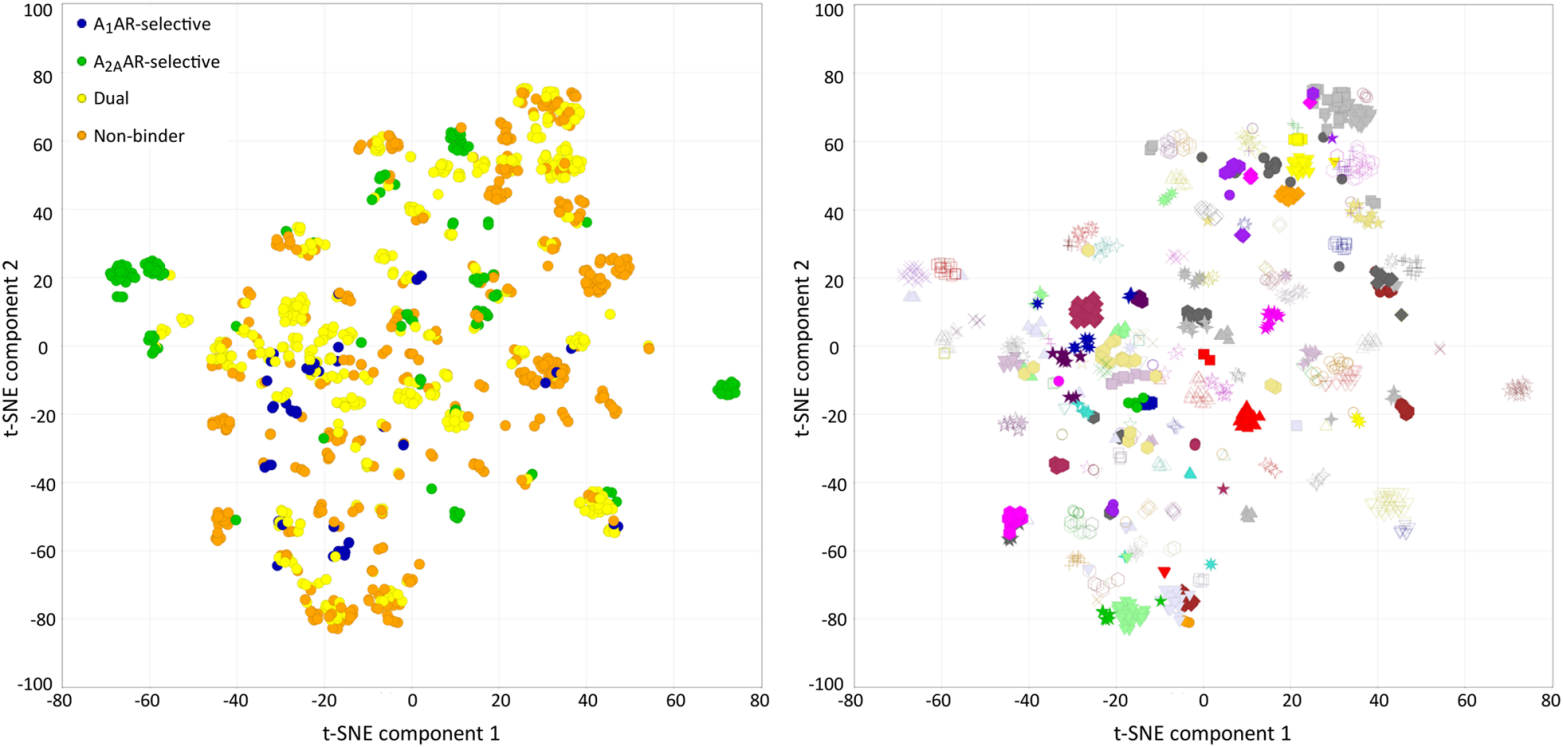
Chemical similarity of compounds of the selectivity classes A_1_AR-selective, A_2A_AR-selective, dual, and non-binders. The chemical similarity is visualized with t-SNE [20] based on FCFP4 fingerprints. **a** The used chemical clusters of the compounds: A_1_AR-selective, A_2A_AR-selective, dual binders, and non-binders. **b** Clusters based on chemical similarity; each color-symbol combination represents a unique cluster (136 clusters in total)

Several QSAR models were created to study selectivity. Firstly, subtype selectivity for the A_1_AR and A_2A_AR was modeled using classification models. Additionally, non-selective compounds (dual binders) and non-binders were modeled. The following four models were constructed: a 2-class model (A_1_AR-selective/A_2A_AR-selective), two 3-class models (A_1_AR-selective/A_2A_AR-selective/dual inhibitors on one hand, and A_1_AR-selective/A_2A_AR-selective/non-binders on the other hand), and a 4-class model (A_1_AR-selective/A_2A_AR-selective/dual/non-binders). All models were validated with fivefold cross-validation, using the same (chemically clustered) test sets per iteration for each model (for details see Methods section). The performance of the 2-class QSAR model was best for predicting A_1_AR and A_2A_AR selectivity (receiver operating characteristic (ROC) 0.87 ± 0.06, and Matthews Correlation Coefficient (MCC) 0.40 ± 0.13). Addition of dual and non-binder classes decreased model performance. ROC decreased to 0.76 ± 0.06 and 0.64 ± 0.09 for the A_1_AR and to 0.62 ± 0.09 and 0.65 ± 0.09 for the A_2A_AR. Likewise, MCC decreases to 0.22 ± 0.15 and 0.00 ± 0.07 for the A_1_AR and to 0.20 ± 0.17 and 0.36 ± 0.12 for the A_2A_AR (Table 2). This indicates that the A_1_AR and A_2A_AR 100-fold selective compounds are sufficiently chemically distinct from each other to be correctly predicted by the model and that they show a clear structure–activity relationship. Conversely, dual and non-binders are suggested to share chemical similarities with both the A_1_AR- and A_2A_AR-selective classes, making it more challenging for the model to differentiate between these classes (Fig. 2). Furthermore, the sensitivity and the positive predictive value (PPV) were consistently higher for A_2A_AR-selective compounds than for A_1_AR-selective compounds, whereas specificity and negative predictive value (NPV) were higher for A_1_AR-selective compounds.

**Table 2:** Performance of selectivity classification models

Classification model	Class	MCC	Sensitivity	Specificity	PPV	NPV	ROC
QSAR 2-class	A_1_AR	0.40 ± 0.13	0.62 ± 0.16	0.76 ± 0.11	0.57 ± 0.12	0.86 ± 0.07	0.87 ± 0.06
	A_2A_AR	0.40 ± 0.13	0.76 ± 0.11	0.62 ± 0.16	0.86 ± 0.07	0.57 ± 0.12	0.87 ± 0.06
QSAR 3-class	A_1_AR	0.22 ± 0.15	0.25 ± 0.15	0.96 ± 0.02	0.31 ± 0.14	0.93 ± 0.02	0.76 ± 0.06
	A_2A_AR	0.20 ± 0.17	0.33 ± 0.16	0.88 ± 0.02	0.35 ± 0.14	0.83 ± 0.04	0.62 ± 0.09
	Dual	0.10 ± 0.09	0.81 ± 0.03	0.29 ± 0.10	0.75 ± 0.02	0.35 ± 0.08	0.58 ± 0.06
QSAR 3-class	A_1_AR	0.00 ± 0.07	0.11 ± 0.05	0.88 ± 0.07	0.10 ± 0.06	0.91 ± 0.02	0.64 ± 0.09
	A_2A_AR	0.36 ± 0.12	0.47 ± 0.14	0.85 ± 0.10	0.59 ± 0.11	0.85 ± 0.02	0.65 ± 0.09
	Non-binder	0.07 ± 0.13	0.67 ± 0.11	0.39 ± 0.12	0.70 ± 0.05	0.37 ± 0.12	0.50 ± 0.09
QSAR 4-class	A_1_AR	0.11 ± 0.10	0.12 ± 0.09	0.97 ± 0.01	0.18 ± 0.11	0.95 ± 0.01	0.70 ± 0.07
	A_2A_AR	0.25 ± 0.16	0.29 ± 0.13	0.94 ± 0.02	0.39 ± 0.16	0.90 ± 0.02	0.67 ± 0.09
	Dual	0.09 ± 0.05	0.51 ± 0.05	0.58 ± 0.08	0.50 ± 0.05	0.60 ± 0.02	0.58 ± 0.05
	Non-binder	0.15 ± 0.06	0.51 ± 0.09	0.64 ± 0.06	0.47 ± 0.04	0.68 ± 0.05	0.57 ± 0.07

Means of fivefold cross-validation with standard error of the mean (SEM). The class indicates the performance for that particular selectivity class: A_1_AR-selective, A_2A_AR-selective, dual (non-selective), and non-binders

*MCC* Matthews Correlation Coefficient, *PPV* positive predictive value, *NPV* negative predictive value, *ROC* receiver operating characteristic

The non-binder class contains compounds that are inactive at both receptors. However, these compounds are not well differentiated from the active classes (A_1_AR-, A_2A_AR-selective, and dual), as is observed by low MCC (0.15 ± 0.06) and poor ROC (0.57 ± 0.07) for the non-binder class. The next section therefore describes bioactivity modeling of the A_1_AR and A_2A_AR in an attempt to categorize non-binders.

### Modeling A_1_AR and A_2A_AR bioactivity using classification and regression QSAR models

The bioactivity of compounds for the A_1_AR and A_2A_AR were modeled with both classification and regression models. Classification models categorize compounds with using a pre-defined threshold (here pActivity ≥ 6.5) as ‘active’ and compounds below that threshold are termed ‘inactive’. The model is trained on these activity classes and provides an activity class for test compounds as well. In contrast, regression models are not trained on classes, but on numerical bioactivity values. The output that is generated from a regression model is a bioactivity value, which can subsequently be assigned to an activity class. As can be observed in Table 1 where the median pActivity for the sets is shown, this value (pActivity 6.5) is applicable for these data sets and was previously also shown to be a relevant threshold leading to balanced classes [15].

Bioactivity classification and regression QSAR models were trained on the A_1_AR/A_2A_AR dataset, the same dataset that was used in the selectivity-classification QSAR models described in the previous section. Additionally, semi-selective compounds were added to increase the amount of training data. These semi-selective compounds have experimental activities for both receptors but do not fit into any of the four selectivity classes (e.g. a compound with pActivity A_1_AR = 7.0, pActivity A_2A_AR = 8.1). However, for bioactivity modeling the selectivity class is irrelevant, and thus these compounds were now included to increase model performance. Additionally, separate bioactivity QSAR models were trained on the A_1_AR and A_2A_AR bioactivity datasets. The validation test sets were composed based on chemical clusters and bioactivity of compounds; each subset contained both actives and inactives. These validation sets were not equal to the aforementioned (selectivity) validation sets as they were used for a different purpose: validation of bioactivity models instead of selectivity models. All bioactivity models were validated using the same cross-validation test sets, regardless of the dataset that was used in training (A_1_AR/A_2A_AR dataset, A_1_AR bioactivity, or A_2A_AR bioactivity). The A_1_AR and A_2A_AR bioactivity datasets contained more data points than the A_1_AR/A_2A_AR dataset as these sets also included single points (bioactivity measured only for one of the two receptors). The single bioactivity points were included in training, but excluded from validation to retain comparability of performance for the different models. Single points that belonged to the same chemical cluster as the data points in the test set were also excluded from training to prevent bias. The regression models show good model quality in training, with a high R^2^ (≥ 0.98) and low RMSE values (≤ 0.14). Unfortunately, when applied on the validation set, performances are lower than expected based on training performance (likely caused by the challenging test set based on chemical clustering). Nevertheless judging the model performance on classification validation metrics a realistic estimation can be made for the predictive performance of the models.

The mean performances of all bioactivity models (based on fivefold cross-validation) were better for the A_2A_AR than for the A_1_AR, with an average ROC difference of 0.12 (Table 3). Furthermore, classification models performed worse than regression models, as indicated by their lower values for enrichment (ROC) and MCC: average difference in ROC for the A_1_AR = 0.20 and A_2A_AR = 0.07, and average difference in MCC for the A_1_AR = 0.07 and A_2A_AR = 0.03. Moreover, the MCC and ROC for A_1_AR bioactivity classification models even indicated performances worse than random (MCC < 0 and ROC < 0.5). The best performing bioactivity models were based on regression, which reached an average performance (ROC score 0.60–0.70) for predicting bioactivities.

**Table 3:** Performances of classification and regression bioactivity models for the A_1_AR and A_2A_AR

Protein	Model training type	Dataset in training	Validation set (only A_1_AR or A_2A_AR compounds, respective of the protein)	MCC	Sensitivity	Specificity	PPV	NPV	ROC
A_1_AR	Classification	A_1_AR compounds in the A_1_AR/A_2A_AR dataset + semi-selective compounds	A_1_AR/A_2A_AR dataset + semi-selective compounds	− 0.09 ± 0.06	0.44 ± 0.09	0.48 ± 0.11	0.46 ± 0.09	0.45 ± 0.06	0.41 ± 0.05
	Classification	A_1_AR bioactivity dataset	A_1_AR/A_2A_AR dataset + semi-selective compounds	− 0.16 ± 0.05	0.39 ± 0.08	0.45 ± 0.10	0.42 ± 0.09	0.41 ± 0.06	0.39 ± 0.04
	Regression	A_1_AR compounds in the A_1_AR/A_2A_AR dataset + semi-selective compounds	A_1_AR/A_2A_AR dataset + semi-selective compounds	0.09 ± 0.04	0.53 ± 0.09	0.56 ± 0.08	0.54 ± 0.09	0.54 ± 0.08	0.61 ± 0.03
	Regression	A_1_AR bioactivity dataset	A_1_AR/A_2A_AR dataset + semi-selective compounds	0.04 ± 0.06	0.46 ± 0.08	0.58 ± 0.08	0.52 ± 0.10	0.52 ± 0.08	0.59 ± 0.05
	Regression	A_1_AR bioactivity dataset	A_1_AR bioactivity dataset	0.06 ± 0.07	0.49 ± 0.07	0.58 ± 0.08	0.53 ± 0.07	0.54 ± 0.06	0.60 ± 0.05
A_2A_AR	Classification	A_2A_AR compounds in the A_1_AR/A_2A_AR dataset + semi-selective compounds	A_1_AR/A_2A_AR dataset + semi-selective compounds	0.11 ± 0.09	0.59 ± 0.10	0.50 ± 0.13	0.73 ± 0.05	0.39 ± 0.05	0.59 ± 0.06
	Classification	A_2A_AR bioactivity dataset	A_1_AR/A_2A_AR dataset + semi-selective compounds	0.16 ± 0.11	0.57 ± 0.12	0.56 ± 0.13	0.75 ± 0.06	0.45 ± 0.09	0.61 ± 0.07
	Regression	A_2A_AR compounds in the A_1_AR/A_2A_AR dataset + semi-selective compounds	A_1_AR/A_2A_AR dataset + semi-selective compounds	0.12 ± 0.10	0.69 ± 0.10	0.40 ± 0.08	0.70 ± 0.04	0.47 ± 0.10	0.64 ± 0.06
	Regression	A_2A_AR bioactivity dataset	A_1_AR/A_2A_AR dataset + semi-selective compounds	0.21 ± 0.07	0.64 ± 0.10	0.56 ± 0.10	0.76 ± 0.04	0.46 ± 0.04	0.69 ± 0.05
	Regression	A_2A_AR bioactivity dataset	A_2A_AR bioactivity dataset	0.19 ± 0.07	0.63 ± 0.11	0.54 ± 0.09	0.70 ± 0.04	0.50 ± 0.05	0.69 ± 0.05

Query compounds were categorized based on post-classification of the bioactivity predictions: predicted pActivity < 6.5 = inactive and predicted pActivity ≥ 6.5 = active

*MCC* Matthews Correlation Coefficient, *PPV* positive predictive value, *NPV* negative predictive value, *ROC* receiver operating characteristic

### Modeling A_1_AR/A_2A_AR subtype selectivity using regression models

The application of the above bioactivity model approach was tested in modeling the selectivity of compounds (i.e. modeling the affinity on the respective receptors and deriving selectivity from that indirectly). As the predicted bioactivities of the two bioactivity models are not correlated with each other, a separate validation was performed to indicate the performance of selectivity predictions. However, the cross-validation sets of the models in Table 3 were clustered based on bioactivity instead of selectivity classes, hence bioactivity models were retrained using differently composed cross-validation sets to justify comparison with a later discussed selectivity-window model. Regression bioactivity models were selected as these outperformed the classification bioactivity models. Moreover, regression was preferred in selectivity modeling as with regression a quantitative value for selectivity can be derived.

Thus, bioactivity regression models were used to predict compound activity for the A_1_AR and A_2A_AR. Models were trained on the A_1_AR/A_2A_AR dataset including additional ‘semi-selective’ compounds. The difference in predicted bioactivity for the two receptors was calculated as selectivity value. Subsequently, selectivity classes (A_1_AR-selective, A_2A_AR-selective, dual) were assigned to the compounds based on the predicted selectivity according to the same categorization rules as used in classification previously. The application of the two combined bioactivity models to deduce selectivity (two-step A_1_AR-A_2A_AR difference model) resulted in models with average performance. Although ROC scores were decent (0.75 ± 0.09 and 0.72 ± 0.15), MCC was poor (0.19 ± 0.16 and 0.28* ± 0.12, *one failed validation) for both the A_1_AR and A_2A_AR, indicating that models were capable of ranking the compounds but less capable of explaining the whole data set (Table 4).

**Table 4:** Performances of selectivity modeling using the two-step A_1_AR-A_2A_AR difference model or the selectivity-window model

Model	Class	MCC	Sensitivity	Specificity	PPV	NPV	ROC
Trained on all double points, tested on all double points (non-binders and semi-selective compounds always true/false negative)							
A_1_AR-A_2A_AR difference	A_1_AR	0.15 ± 0.13	0.17 ± 0.14	0.99 ± 0.00	0.18 ± 0.12	0.98 ± 0.01	0.76 ± 0.09
	A_2A_AR	0.11 ± 0.07	0.19 ± 0.09	0.94 ± 0.02	0.15 ± 0.07	0.94 ± 0.01	0.67 ± 0.14
	Dual	0.26 ± 0.05	0.76 ± 0.02	0.50 ± 0.07	0.68 ± 0.02	0.59 ± 0.03	0.66 ± 0.02
Selectivity-window	A_1_AR	0.07** ± 0.09	0.030.03	0.99 ± 0.00	0.10** ± 0.10	0.97 ± 0.00	0.87 ± 0.03
	A_2A_AR	0.22 ± 0.12	0.15 ± 0.09	0.99 ± 0.00	0.42 ± 0.18	0.94 ± 0.01	0.74 ± 0.07
	Dual	0.36 ± 0.06	0.85 ± 0.03	0.48 ± 0.03	0.69 ± 0.01	0.70 ± 0.06	0.70 ± 0.02
Trained on all double points, tested on only A_1_AR, A_2A_AR, and dual class							
A_1_AR-A_2A_AR difference	A_1_AR	0.19 ± 0.16	0.17 ± 0.14	0.99 ± 0.00	0.28 ± 0.18	0.96 ± 0.01	0.75 ± 0.09
	A_2A_AR	0.28* ± 0.12	0.19 ± 0.09	0.98 ± 0.01	0.48* ± 0.15	0.91 ± 0.01	0.72 ± 0.15
	Dual	0.25 ± 0.07	0.76 ± 0.02	0.57 ± 0.11	0.91 ± 0.02	0.28 ± 0.04	0.70 ± 0.05
Selectivity-window	A_1_AR	0.17** ± 0.23	0.03 ± 0.03	0.99 ± 0.01	0.50** ± 0.50	0.92 ± 0.01	0.88 ± 0.04
	A_2A_AR	0.32* ± 0.15	0.15 ± 0.09	1.00 ± 0.00	0.75* ± 0.25	0.82 ± 0.03	0.80 ± 0.07
	Dual	0.33 ± 0.11	0.84 ± 0.04	0.46 ± 0.06	0.80 ± 0.02	0.55 ± 0.10	0.66 ± 0.04
Trained on only A_1_AR, A_2A_AR, and dual class, tested on only A_1_AR, A_2A_AR, and dual class							
Selectivity-window	A_1_AR	− 0.05*** ± 0.00	0.00 ± 0.00	0.99 ± 0.01	0.00*** ± 0.00	0.92 ± 0.01	0.81 ± 0.05
	A_2A_AR	0.23 ± 0.18	0.25 ± 0.12	0.94 ± 0.03	0.43 ± 0.22	0.83 ± 0.03	0.66 ± 0.11
	Dual	0.04 ± 0.12	0.69 ± 0.05	0.35 ± 0.09	0.73 ± 0.03	0.32 ± 0.09	0.53 ± 0.08

The class indicates the performance for that particular selectivity class: A_1_AR-selective, A_2A_AR-selective, and dual (non-selective). The query compounds were categorized based on post-classification of the selectivity predictions: A_1_AR-selective when pActivity difference ≥ 2, A_2A_AR-selective when pActivity difference ≤ − 2, and dual binder when pActivity difference ≥ − 1 and ≤ 1

*MCC* Matthews Correlation Coefficient, *PPV* positive predictive value, *NPV* negative predictive value, *ROC* receiver operating characteristic

*1/5 folds failed, **3/5 folds failed, ***4/5 folds failed

Continuing our single model approach to predict selectivity we explored the usage of regression for a selectivity-window model rather than classification. In contrast to the two-step A_1_AR-A_2A_AR difference model (regression), the single model to predict selectivity between the A_1_AR and A_2A_AR was based on the difference in affinity rather than the prediction of bioactivity and calculation of the resulting selectivity. The regression model was trained directly on the difference in bioactivity for both receptors (pActivity A_1_AR − pActivity A_2A_AR = selectivity-window) and predicts a quantitative score for the degree of selectivity of a compound (difference in pActivity). A positive score indicates A_1_AR-selectivity, a negative score A_2A_AR-selectivity, and a score close to zero indicates dual binders. The model was evaluated based on the ROC score and classification metrics (MCC, sensitivity, specificity, PPV, and NPV). The rules for classification of the A_1_AR-, A_2A_AR-selective, and dual binders were derived from the thresholds applied in the selectivity classification models: A_1_AR ≥ 100-fold selective equals pActivity difference ≥ 2, A_2A_AR ≥ 100-fold selective equals pActivity difference ≤ − 2, and for dual binders (≤ tenfold selective) pActivity difference ≥ − 1 and ≤ 1.

The selectivity-window regression model was trained on the same data (A_1_AR/A_2A_AR dataset and semi-selective compounds) as the two-step A_1_AR-A_2A_AR difference model described above in which selectivity was deducted from two separate bioactivity models. The selectivity-window outperformed the two-step A_1_AR-A_2A_AR difference model with increased ROC values for selectivity classes A_1_AR- and A_2A_AR-selective (ROC increase 0.07–0.13) (Table 4).

**Fig. 3:**
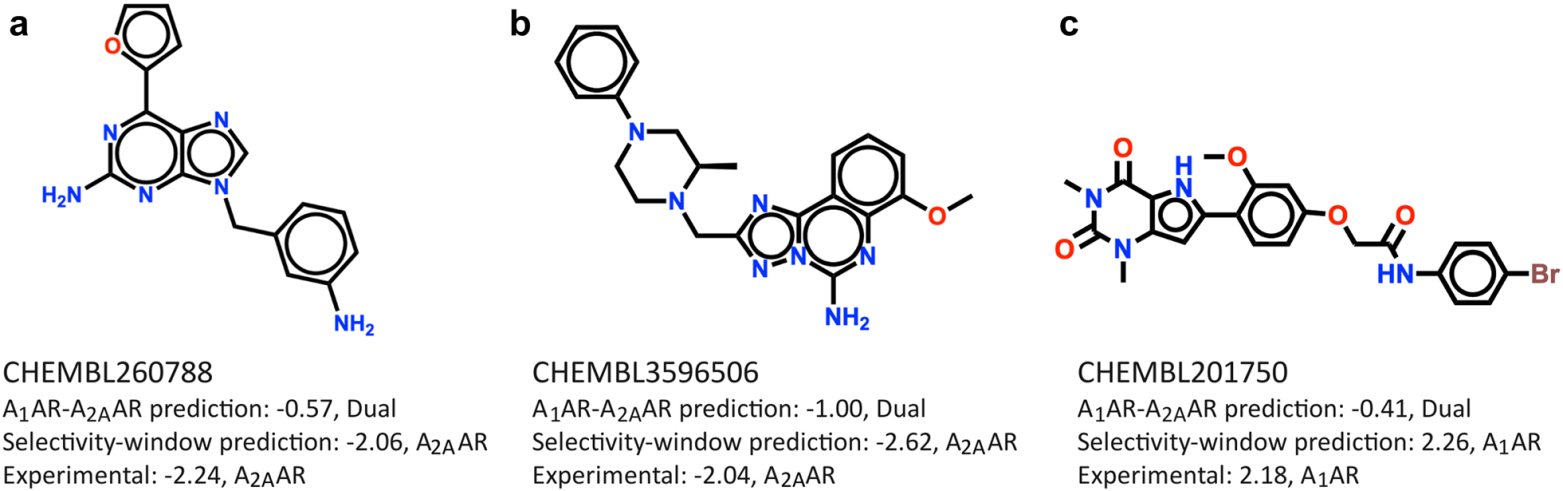
Chemical structures of compounds with predictions by different selectivity models. The compounds were wrongly predicted with the two-step A_1_AR-A_2A_AR model and correctly predicted with the selectivity-window model. Predictions are indicated as: predicted A_1_AR-selective (A_1_AR), A_2A_AR-selective (A_2A_AR), and as dual binder (Dual) for ligands CHEMBL260788 (**a**), CHEMBL3596506 (**b**), and CHEMBL201750 (**c**)

Figure 3 shows example compounds that were misclassified with the two-step A_1_AR-A_2A_AR difference model, but were correctly predicted using the selectivity-window model. The similarity (Tanimoto FCFP4) between the mispredicted compounds by the two-step A_1_AR-A_2A_AR difference model was 0.25, whereas the similarity within wrongly predicted compounds by the selectivity-window model was 0.58. This indicates that the two-step A_1_AR-A_2A_AR difference model is challenged by selectivity prediction of more diverse compounds and the selectivity-window model underperforms on specific chemical scaffolds. The most frequently mispredicted scaffold by the selectivity-window model was *N*-(2-(furan-2-yl)-6-(1*H*-pyrazol-1-yl)pyrimidin-4-yl)-2-phenoxyacetamide (see Additional file 2).

It should be noted that for the A_1_AR and A_2A_AR predictions MCC and PPV could not always be calculated in cross-validation, resulting in failed cross-validation folds, or iterations. This is explained by the lack of true/false positives in the particular cross-validation fold: PPV cannot be calculated if there are no positives, MCC cannot be calculated without a PPV.

The selectivity-window model performed better than the three-class selectivity classification model validated earlier (Table 2). It was tested whether this increased performance was a result from the increased amount of data as the selectivity-window model included additional semi-selective compounds and non-binders, which the three-class selectivity classification model necessarily lacked as the affinity difference was not large enough to meet the classification cut-off. When these additional data points were excluded from the selectivity-window model, the ROC dropped from 0.78 to 0.67 (average over classes), which is comparable with the ROC of 0.65 of the three-class selectivity classification model. This observation clearly confirms a direct link between model quality and data availability and shows that the increased performance of the selectivity-window model is attributed to additional data points. Hence it is advantageous to use continuous models in selectivity modeling as in this case more data can be included. In addition to the benefit of increased data availability, continuous selectivity models also provide the ability to calculate a selectivity ratio as opposed to the class only. This selectivity ratio indicates the degree of selectivity and therefore cannot only identify selective compounds, but can also differentiate highly selective compounds from weakly selective compounds.

**Fig. 4:**
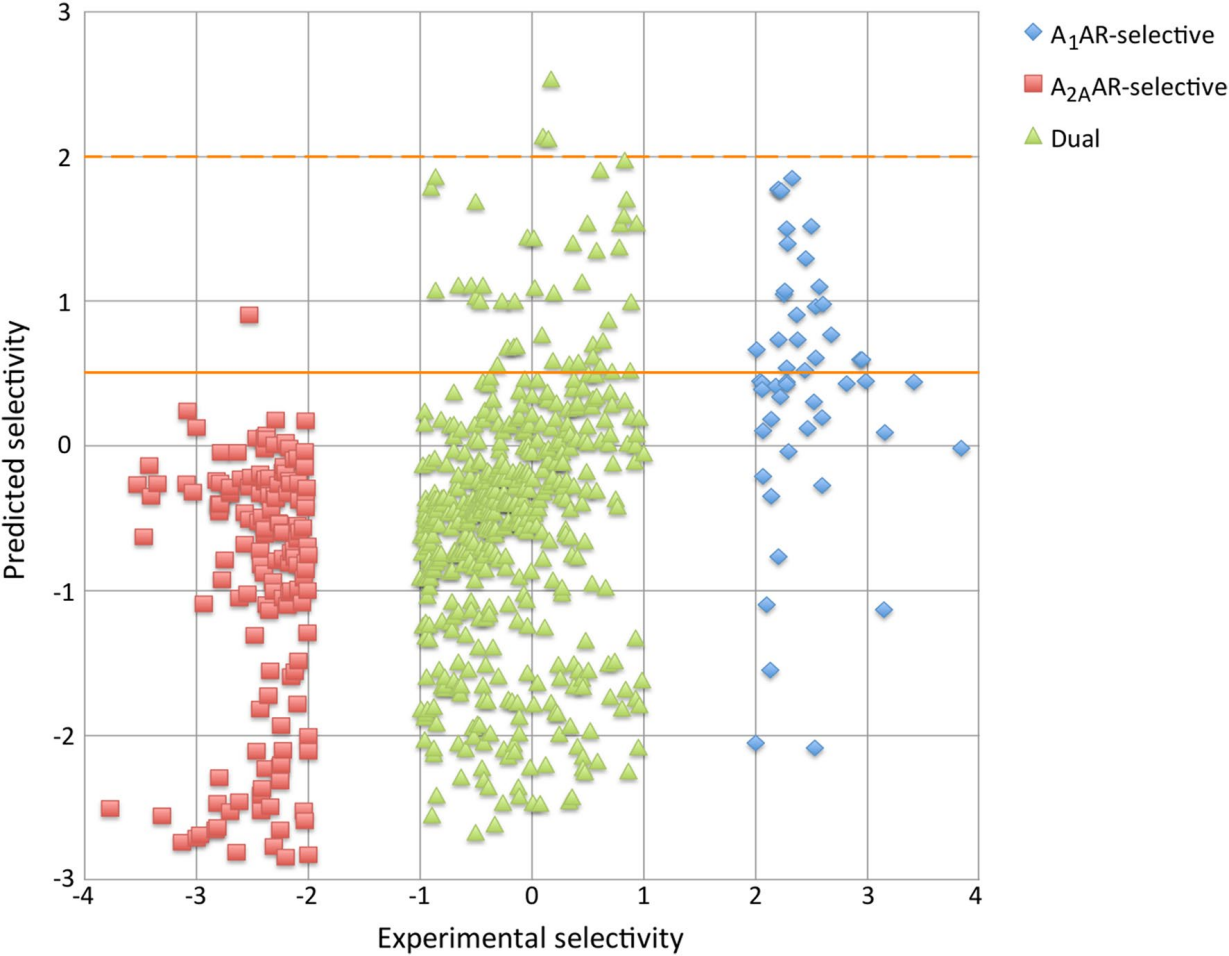
Relationship between experimental selectivity versus predicted selectivity. Predicted selectivity values shown for the selectivity-window model. A_1_AR-selective classification thresholds shown as orange lines (dotted = old threshold, solid = new threshold)

Remarkably, metrics based on classification (MCC, sensitivity, specificity, PPV, and NPV) for the selectivity-window model (without non-binders and semi-selective compounds) (Table 4) are lower for the A_1_AR-selective compounds and dual binders than metrics of the 3-class classification model (both trained on the same data) (Table 2), whereas ROC scores are comparable or higher. Therefore, the predictions of the selectivity-window model were compared with the experimentally measured selectivity values (Fig. 4). It was observed that the A_1_AR-selective compounds have consistently lower selectivity-window predictions than the experimental selectivity values. As a result, fewer compounds reached the A_1_AR-selective classification threshold, decreasing the number of the A_1_AR-selective positives drastically. From the 50 A_1_AR-selective compounds, none reached the threshold. Of all predictions, only three compounds reached the A_1_AR-selective threshold, which were dual binders instead of the A_1_AR-selective compounds. This deficiency of predicted positives explains the failed cross-validation calculations for the A_1_AR-selective class.

To compensate, classification validation metrics were re-calculated post hoc using classification thresholds that were adapted to compensate for the generalization of selectivity for A_1_AR-selective compounds. Compounds were deemed A_1_AR-selective when the selectivity-window ≥ 0.5, A_2A_AR-selective when the selectivity-window ≤ − 2 (unchanged), and dual binder when the selectivity-window ≥ − 1 and  < 0.5. Using the new thresholds, values of the metrics for the A_1_AR-selectivity predictions improved: MCC 0.31 ± 0.10, sensitivity 0.45 ± 0.12, specificity 0.91 ± 0.02, PPV 0.32 ± 0.06, and NPV 0.95 ± 0.01, indicating that the revised threshold improves the categorization of the A_1_AR-selective compounds. Not all A_1_AR-selective compounds were correctly categorized but the post hoc optimized threshold was considered adequate, as lowering the A_1_AR-selective threshold further would increase sensitivity (by categorization of more compounds as A_1_AR-selective), but would also decrease PPV. Here, the correctness of predictions was prioritized over the number of predicted active compounds; hence PPV was prioritized over sensitivity.

### Removal of non-binders to enhance performance

Although the selectivity-window model differentiates between the A_1_AR-, A_2A_AR-selective compounds, and dual binders, the model does not consider potential inactivity of compounds. Consequently, non-binders cannot be filtered using this model. Therefore, a consensus approach of statistical modeling and structure-based docking was applied to identify and exclude non-binders.

Bioactivity regression models described above for the A_1_AR and A_2A_AR were combined with docking of the A_1_AR/A_2A_AR dataset and semi-selective compounds into crystal structures of both proteins. Bioactivity predictions for the A_1_AR and A_2A_AR, and selectivity-window predictions, were derived for the entire A_1_AR/A_2A_AR dataset and semi-selective compounds by assembling the predictions made during fivefold cross-validation of the previously trained regression models. Compounds were docked into crystal structures of the A_1_AR (PDB: 5UEN) [10] and the A_2A_AR (PDB: 5OLZ) [21], which resulted in docking scores for both receptors. Compounds were assigned a separate bioactivity label for the A_1_AR and A_2A_AR: compounds in the A_1_AR were labeled ‘active’ when predicted pActivity ≥ 7 and docking score ≤ − 9. Compounds in the A_2A_AR were labeled ‘active’ when predicted pActivity ≥ 7 and docking score ≤ − 10.

**Fig. 5:**
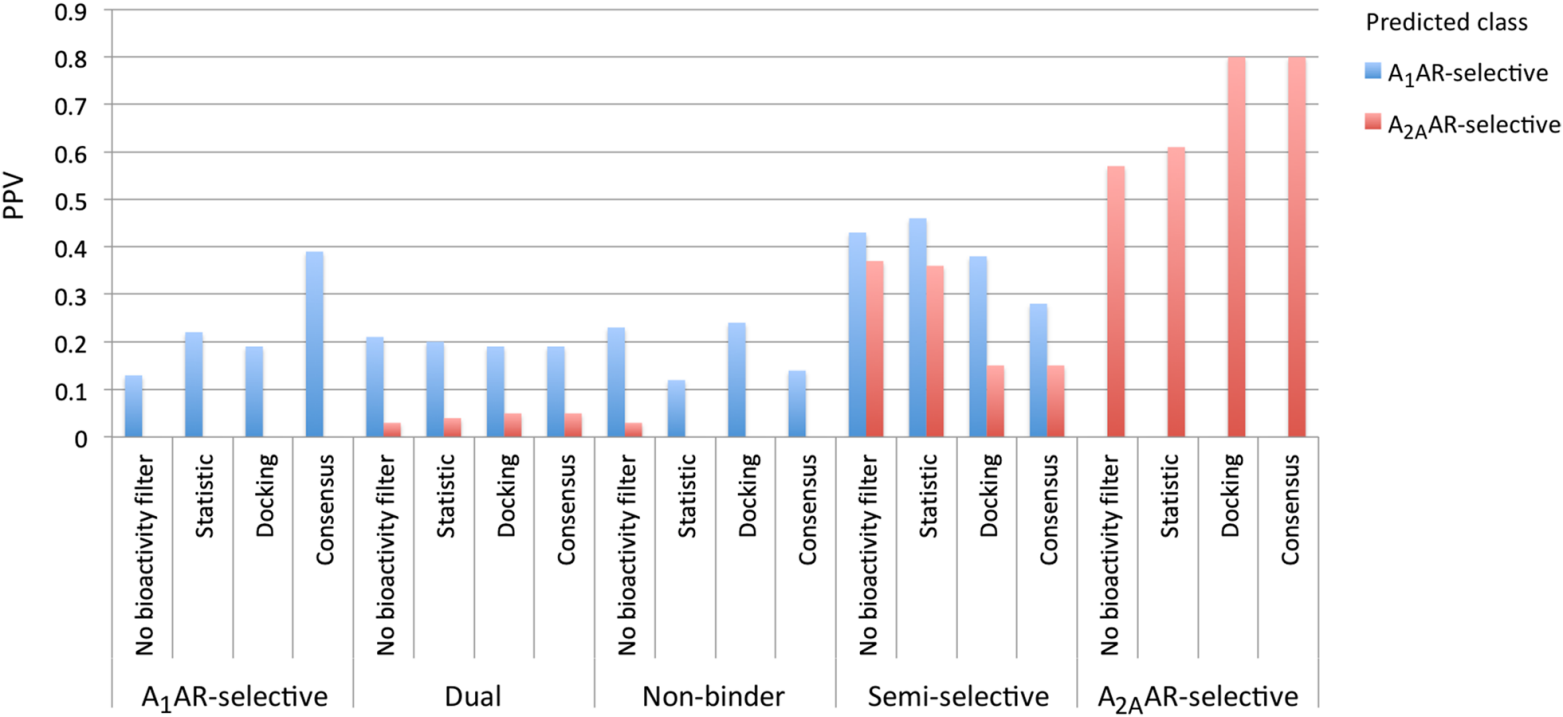
Positive predictive value (PPV) of compounds predicted to be A_1_AR- or A_2A_AR-selective. The PPV depicts the number of experimentally validated selective compounds divided by the total number of predicted selective compounds. PPVs are shown when different filters are applied: no bioactivity filter, statistical bioactivity, bioactivity based on docking score, and consensus bioactivity (statistical bioactivity and structure-based docking)

Compounds with predicted selectivity-window ≥ 0.5 or ≤ − 2, corresponding with A_1_AR- and A_2A_AR-selective, were subsequently filtered using the consensus bioactivity filter (Fig. 5). The PPV for A_1_AR-selective compounds drastically increases from 0.13 to 0.39 when the selectivity-window predictions were filtered using the consensus bioactivity filter for the A_1_AR. The A_2A_AR bioactivity filtering also increased the PPV of A_2A_AR-selective compounds; here docking and consensus filtering performed equally well (PPV A_2A_AR-selective: 0.80).

**Fig. 6:**
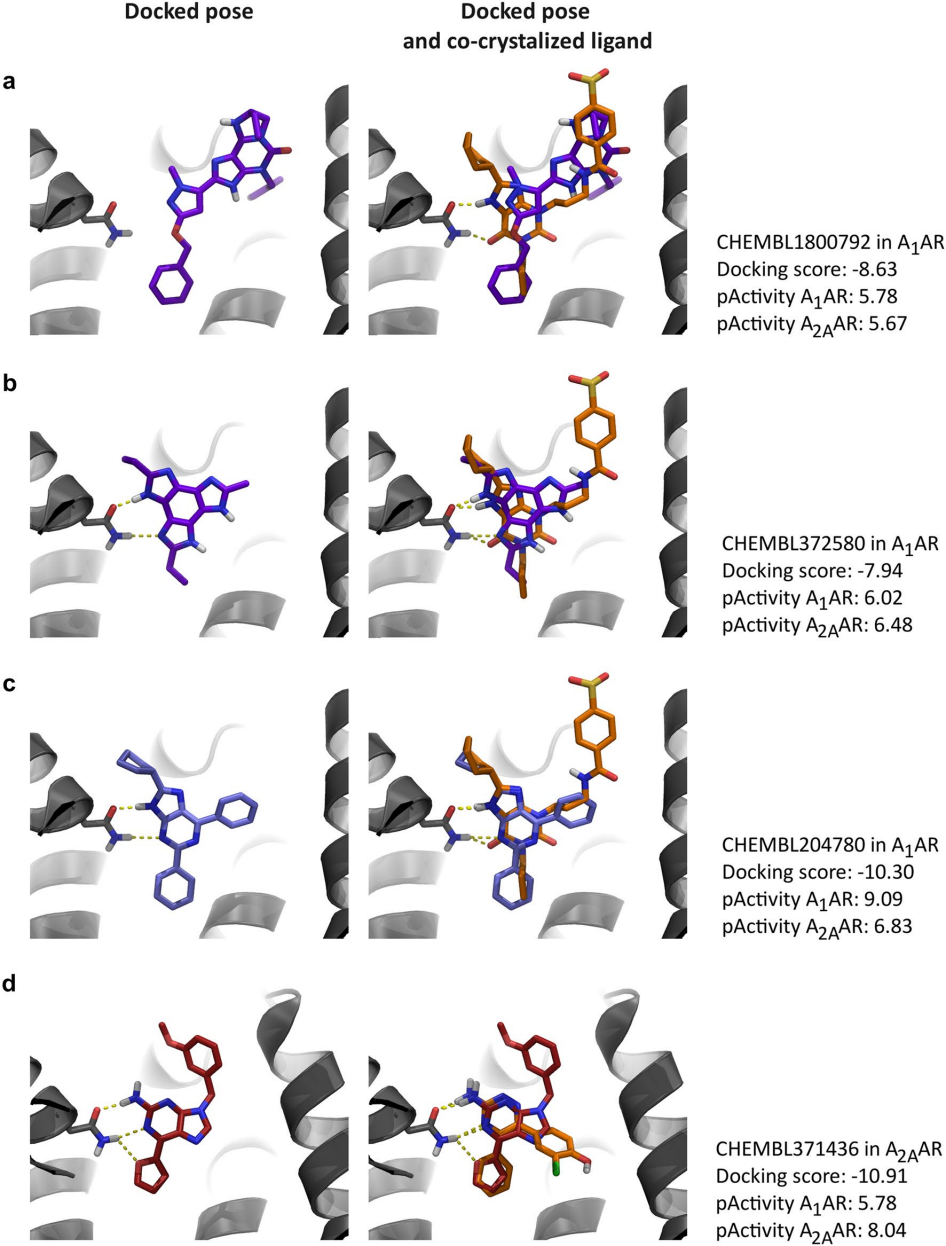
Docked poses of compounds in their corresponding targets. Poses of two non-binders in the A_1_AR (CHEMBL1800792 in **a** and CHEMBL372580 in **b**), an A_1_AR-selective compound (CHEMBL204780 in **c**), and A_2A_AR-selective compound (CHEMBL371436 in **d**). Docked poses are compared to the co-crystalized ligands shown in orange. Hydrogen bonds between ligands and Asn^6.55^ are shown in yellow

Non-binders that were not removed using the statistical filter only, but were filtered when the consensus approach was used, were inspected in the crystal structure of the A_1_AR. Some non-binders (e.g., CHEMBL1800792) did not adapt a favorable conformation when docked into the A_1_AR (Fig. 6). Moreover, an interaction with pocket residue Asn^6.55^ (Ballesteros-Weinstein numbering) was frequently not observed. This is an essential missing element, as interaction with this residue has shown to be important for ligand binding to the A_1_AR and A_2A_AR [11, 22]. However, some non-binders were able to make this interaction (CHEMBL372580), but nevertheless had a docking score that did not reach the set threshold (docking score ≤ − 9). The poses of the non-binders were compared to those of an A_1_AR-selective (CHEMBL207824) and an A_2A_AR-selective compound (CHEMBL371436). Both selective compounds adapt a conformation that is able to make an interaction with residue Asn^6.55^. Furthermore, the poses also constitute the same aromatic interactions (with Phe171^EL2^ in the A_1_AR and with Phe168^EL2^ and His250^6.52^ in the A_2A_AR) as the co-crystalized ligands and adapt a similar scaffold orientation. Finally, the poses of the selective ligands have favorable docking scores (− 10.30 and − 10.91, respectively), supporting that these compounds are binders for the A_1_AR or A_2A_AR.

### Validation of the selectivity-window model on an external set

The predictive selectivity-window model (trained on A_1_AR/A_2A_AR dataset and semi-selective compounds) was challenged to predict the selectivity of compounds from an external validation set. This set contained 1482 compounds of which a dose–response bioactivity value (K_i_/IC_50_/EC_50_/K_d_) was known for at least one of the two receptors. If an accurate bioactivity value was available for both receptors, the compound was classified according to prior rules applied in this study. However, if an accurate bioactivity value for only one receptor was known, a less accurate bioactivity measurement (inhibition as percentage displacement/efficacy/change) was used to identify inactivity for the missing receptor. The low accuracy of the bioactivity values makes this data less suitable for model training on the quantitative difference between activity on the two receptors, but suitable for classification validation. A pChEMBL value of < 4.5 or inhibition threshold of ≤ 50% (at 10 μM) was used to label inactive compounds, whereas a pChEMBL value of ≥ 6.5 was used to indicate active compounds. The selectivity-window model (see Additional file 3) was applied to the compounds in the external validation set, providing them all with a predicted selectivity score and, subsequently, a selectivity class. The validation encompassed all selectivity classes: A_1_AR- and A_2A_AR-selective compounds, dual binders and non-binders. Since the selectivity-window model has no threshold for non-binders, non-binders were always considered either as true or false negative (never false/true positive).

Without filtering inactives, the selectivity-window model performed average in the prediction of the A_1_AR-selective compounds (ROC 0.75) and A_2A_AR-selective compounds (ROC 0.66) in the external validation set (Table 5). However, application of the consensus bioactivity filter resulted in an increase of the classification enrichment of the A_1_AR- and A_2A_AR-selective compounds. Although the ROC for A_1_AR-selective compounds decreased after applying the bioactivity filter, PPV, and thus the fraction of true A_1_AR-selective compounds compared to all predicted A_1_AR compounds, increased from 0.12 to 0.21. In addition, MCC increased slightly from 0.13 to 0.18. Inspection of the compounds showed that all non-binders were removed after filtering the selectivity-window predictions with the consensus bioactivity filter. The decrease in ROC for the A_1_AR-selective class was thus caused by the presence of dual binders only. Remarkably, sensitivity for the A_1_AR- and A_2A_AR-selective compounds was both 1.00 (100%), whereas sensitivity for dual binders was 0.00 (0%). Although dual compounds were present in the set that was filtered with the selectivity-window model, these compounds were wrongly categorized as either A_1_AR- or A_2A_AR-selective. The predicted dual binders prior to bioactivity filtering were, in fact, non-binders. However, these non-binders were correctly filtered out using the bioactivity filter, leaving the dual binder class without positive-predicted compounds. Note that the results do not specify dual binder enrichment, as the bioactivity predictions encompassed only compounds predicted to be A_1_AR- or A_2A_AR-selective.

**Table 5:** Performance of the selectivity-window model on an external validation set

Model	Class	MCC	Sensitivity	Specificity	PPV	NPV	ROC
Selectivity-window	A_1_AR	0.13	0.39	0.83	0.12	0.96	0.75
	A_2A_AR	0.40	0.24	1.00	0.70	0.97	0.66
	Dual	0.02	0.81	0.21	0.64	0.39	0.37
Selectivity-window and bioactivity filtered	A_1_AR	0.18	1.00	0.16	0.21	1.00	0.66
	A_2A_AR	0.88	1.00	0.97	0.80	1.00	0.98
	Dual	–	0.00	1.00	–	0.28	0.72

The query compounds were categorized based on post hoc optimized classification of the selectivity predictions: A_1_AR-selective when selectivity-window ≥ 0.5, A_2A_AR-selective when selectivity-window ≤ − 2, and dual binder when selectivity-window ≥ − 1 and < 0.5

*MCC* Matthews Correlation Coefficient, *PPV* positive predictive value, *NPV* negative predictive value, *ROC* receiver operating characteristic

## Discussion

While QSAR models are widely applied in bioactivity modeling, they can also effectively be used in selectivity modeling. However, modeling of selectivity requires a substantial amount of data, as activities for more than one protein have to be measured. The amount of data that is available influences the performance of the selectivity model as was observed for the performances of the selectivity-window models when trained on limited data. To increase the amount of data that is sufficient for selectivity modeling continuous regression models can be applied instead of classification models. With regression not only compounds that belong to a defined selectivity class can be included, but also compounds of which there is some selectivity but not large enough to fit into a class. Another benefit of regression is that the degree of selectivity can be provided in addition to the selectivity class of a compound.

Multiple QSAR regression models to derive selectivity for a panel of kinases were used by Sciabola et al. [23]. First, regression bioactivity models were trained for every kinase in the panel. Next, bioactivity patterns were predicted for a set of compounds against all kinases, from which subsequently selectivity was derived. To compare, we repeated a similar approach was repeated by us in the current work. However, we also introduce the selectivity-window model, which is a direct implementation of selectivity. We show that this approach outperformed models that predicted selectivity indirectly by using separate bioactivity models. Even though separate bioactivity models can include more data since compounds measured for just one protein can be considered, this approach did not increase model performance enough to outperform the selectivity-window model.

Nevertheless, an advantage of using separate bioactivity models to deduce selectivity from is that additional proteins can be added easily: the selectivity between the added protein and existing proteins can quickly be deduced from the results of the added bioactivity model. In selectivity-window modeling multiple models need to be trained to predict selectivities of compounds against a panel of targets: one model for every target-target combination. However, while using separate bioactivity models can be more convenient, selectivity-window modeling may yield more accurate predictions. It should also be noted that automatically generating these models using scripting can be considered trivial. Therefore, when sufficient selectivity data is available it is worthwhile to apply selectivity-window modeling.

The higher accuracy of selectivity-window modeling compared to using multiple bioactivity models is suggested to be influenced by the higher quality of the data used in selectivity-window modeling. In selectivity-window modeling, selectivity is predicted based on data from biological experiments. Although biological experiments are susceptible to error (on average an error of 0.6 log units [24]), this data is more reliable than data derived from statistical models whose error by definition should be higher than the error of the data they were trained on. In practice the error of statistical models (and hence the error of predictions) varies around 0.5–1.0 log units [25, 26]. While this error may accumulate with the experimental error, there is also the possibility that modeling can reduce part of the experimental error. An additional study is required to reveal how the modeling error behaves in combination with the experimental error.

The selectivity-window model by itself is not capable of distinguishing actives from inactives as it is trained on the difference only, which is different from the affinity. However, separate bioactivity models can be applied to filter potentially selective compounds from inactives, or non-binders. A study by Zhao et al., where subtype selectivity between epigenetic targets HDAC1 and HDAC6 was modeled by classification of selectivity, utilizes a comparable approach by first predicting selectivity, followed by bioactivity [27]. However, the selectivity model in that study is incapable of predicting the degree of selectivity as a classification model was used. Furthermore, only statistical models were used by the authors to predict bioactivity of compounds. In the current study it was observed that implementation of statistical bioactivity models only increased the enrichment of selective compounds slightly. In contrast, addition of structure-based docking scores increased the enrichment of selective compounds substantially for both the A_1_AR and A_2A_AR. Moreover, structure-based docking performed equally well as the consensus model (statistical bioactivity and docking) for A_2A_AR-selective enrichment.

## Conclusion

We demonstrated that continuous QSAR models can be applied to model selectivity on the A_1_AR and A_2A_AR. The selectivity-window model, which was trained directly on the difference in affinity between both receptors, outperformed a two-step A_1_AR-A_2A_AR selectivity model. In the two-step model, which is generally applied in selectivity modeling, selectivity predictions are derived indirectly by calculation of the difference between bioactivity predictions that resulted from two separate models. Even though the separate bioactivity models included more data, the performance did not increase enough to outperform our selectivity-window model. Furthermore, a combination of statistical bioactivity models and structure-based docking contributed to the enrichment of selective compounds and can be used to exclude non-binders (which are not predicted accurately when directly predicting selectivity). In summary, we demonstrated that accurate selectivity predictions can be made for the A_1_ and A_2A_ adenosine receptors by combining the selectivity-window model and consensus bioactivity modeling. This method can easily be applied to other protein targets (e.g., kinases) as well, provided sufficient data is available.

## Methods

### Training/test datasets

The dataset was compiled from publicly available data derived from the ChEMBL database [16, 28] (version 23) and in-house data from Leiden University (Leiden, The Netherlands). Compounds with experimental activities were collected for the human A_1_AR (P30542) and human A_2A_AR (P29274). The data derived from ChEMBL was filtered on confidence score 7 and 9, and a pChEMBL value ≥ 4. In-house data was filtered similarly: activity (K_i_/IC_50_/EC_50_) ≤ 10^−4^ M. K_i_ values were prioritized over IC_50_ or EC_50_ values. Thus, for duplicates, when more than one type was available for a given compound-receptor pair, K_i_ values were kept and IC_50_ and EC_50_ values were discarded. The mean value was taken when multiple bioactivity values of the same type were reported for a given compound-receptor pair (e.g., mean of multiple K_i_ values for the same compound). The standardized activity values are reported as pActivity values.

An antagonist-focused dataset was compiled from the filtered data by removing compounds with a ribose or dicyanopyridine scaffold. From this antagonist-focused dataset an A_1_AR/A_2A_AR dataset that contained only compounds with activities measured on both the A_1_AR and A_2A_AR was derived. The compounds were assigned to the A_1_AR/A_2A_AR dataset after they were categorized into one of the following five classes: non-binders when pActivity for both the A_1_AR and A_2A_AR < 6.5, A_1_AR-selective (pActivity ≥ 6.5 for the A_1_AR and activity compared to the A_2A_AR ≥ 100-fold), A_2A_AR-selective (pActivity ≥ 6.5 for the A_2A_AR and activity compared to the A_1_AR ≥ 100-fold), and dual binders (pActivity ≥ 6.5 for both the A_1_AR/A_2A_AR and activity difference ≤ 10-fold). Compounds with experimental bioactivities for both the A_1_AR and A_2A_AR, but that did not fit into any of the classes of the A_1_AR/A_2A_AR dataset, were termed “semi-selective” compounds (855 bioactivities). The antagonist-focused dataset contained 5897 activities, the A_1_AR/A_2A_AR dataset included 1106 compounds, of which 50 A_1_AR-selective and 146 A_2A_AR-selective. Additionally, the antagonist-focused dataset was split into two datasets for bioactivity modeling: the A_1_AR bioactivity dataset (2774 compounds) and the A_2A_AR bioactivity dataset (3123 compounds).

### T-distributed stochastic neighbor embedding (t-SNE)

The chemical similarity of the A_1_AR/A_2A_AR dataset was plotted using t-SNE [20]. Compounds were described using FCFP4 fingerprints (fixed-length array of bits 2024). Two dimensions were calculated: t-SNE component 1 and t-SNE component 2. The settings were as follows: maximum number of iterations 5000, theta 0, perplexity 30, momentum 0.5, final momentum 0.8, and learning rate 10. Additionally a t-SNE was conducted showing the distribution of chemically clustered compounds using affinity propagation (FCFP4) [29].

### External validation set

An external validation set was created by using compounds that had been newly added in ChEMBL version 24 and 25. Furthermore, compounds with confidence score 6 and 8 from previous ChEMBL versions were added. Additionally, less accurate bioactivity measurements (e.g., % displacement) were used to identify inactives. These less accurate bioactivities included bioactivities measured as percentage displacement, efficacy and change. If a pChEMBL value was known for both receptors, the compounds were categorized into the selectivity classes A_1_AR-selective, A_2A_AR-selective, dual binder, and non-binder according to the same rules as used for the A_1_AR/A_2A_AR dataset. If a pChEMBL value was known for only one of the two receptors, less accurate measurements (displacement/efficacy/change) were used to identify if the compound was marked as inactive for the other receptor. Subsequently, a selectivity class could be assigned. A pChEMBL value of < 4.5 or inhibition threshold of ≤ 50% (at 10 μM) was used to label compounds as inactive and a pChEMBL value of ≥ 6.5 was used to identify active compounds. Subsequently the selectivity class was derived from these bioactivity classes: A_1_AR-selective if active on the A_1_AR and inactive on the A_2A_AR, A_2A_AR-selective if inactive on the A_1_AR and active on the A_2A_AR, and non-binder if inactive on both the A_1_AR and A_2A_AR. Again, compounds with ribose and dicyanopyridine scaffolds were excluded, resulting in an external validation set of 1482 compounds.

### Machine learning

QSAR bioactivity and selectivity models were constructed using the R XGBoost model component in Pipeline Pilot (version 18.1.0.1604) [30]. The following settings were applied for both classification and continuous models: maximum number of trees 1000, learning rate 0.3, maximum depth 7, data fraction 1.0, and descriptor fraction 0.7. Compound descriptors were calculated within the component and included ALogP, molecular weight, number of H-donors, number of H-acceptors, number of rotatable bonds, number of atoms, number of (aromatic) rings, and FCFP6 fingerprints (fixed-length array of bits 2024). The workflow to train the selectivity-window model in Pipeline Pilot is included in Additional file 3 (trained with the dataset in Additional file 1). A comparable model training protocol in KNIME [31] is added in Additional file 4 (trained with Additional file 5).

### Cross-validation

The models were validated with fivefold cross-validation where they were trained on 80% and tested on 20% of the dataset. The A_1_AR/A_2A_AR dataset was split into five subsets that each contained all four classes (A_1_AR-selective/A_2A_AR-selective/dual/non-binder) and the A_1_AR bioactivity dataset and A_2A_AR bioactivity dataset were both separated into five subsets considering an active/inactive distribution (Table 6). This consideration of class-distribution ensured that every subset contained each (bioactivity) class, which allows for balanced model training and validation. Chemical similarity of compounds was also considered; the A_1_AR/A_2A_AR dataset and bioactivity datasets were each split into five subsets with every set covering different chemical structures. In order to create five chemically distinct subsets, each selectivity/activity class was clustered into ten clusters with the cluster molecules component in Pipeline Pilot (based on FCFP4). Subsequently, the smallest and largest clusters were combined into one group. This was done recurrently until all clusters were divided into five groups with every group containing 2 clusters per class. Finally, the resulting groups of each selectivity/activity class were distributed equally, resulting in five chemically distinct subsets comprising all selectivity/activity classes. The model performances were evaluated using the following metrics: MCC, Matthews Correlation Coefficient; sensitivity; specificity; PPV, Positive Predictive Value; NPV, Negative Predictive Value; and ROC, receiver operating characteristic [32, 33]. The (traditionally classification) metrics MCC, sensitivity, specificity, PPV, and NPV, were either derived from the classification models directly, or calculated from the output of the regression models.

**Table 6:** Properties of different subsets in the A_1_AR/A_2A_AR dataset, A_1_AR bioactivity dataset, and A_2A_AR bioactivity dataset

Dataset	Subset	Chemical similarity (Tanimoto FCFP4)	Number of compounds	Number of actives (pActivity ≥ 6.5)	Number of inactives (pActivity < 6.5)	Number of A_1_AR-selectives	Number of A_2A_AR-selectives	Number of dual binders	Number of non-binders
A_1_AR/A_2A_AR dataset	1	0.26	362	n.a.	n.a.	11	52	146	153
	2	0.21	261	n.a.	n.a.	11	38	111	101
	3	0.20	171	n.a.	n.a.	12	21	86	52
	4	0.21	156	n.a.	n.a.	10	20	70	56
	5	0.19	156	n.a.	n.a.	6	15	66	69
A_1_AR bioactivity dataset	1	0.23	718	501	217	n.a.	n.a.	n.a.	n.a.
	2	0.20	551	304	247	n.a.	n.a.	n.a.	n.a.
	3	0.20	524	281	243	n.a.	n.a.	n.a.	n.a.
	4	0.19	477	261	216	n.a.	n.a.	n.a.	n.a.
	5	0.19	504	306	198	n.a.	n.a.	n.a.	n.a.
A_2A_AR bioactivity dataset	1	0.25	1463	994	469	n.a.	n.a.	n.a.	n.a.
	2	0.22	467	263	204	n.a.	n.a.	n.a.	n.a.
	3	0.23	460	312	148	n.a.	n.a.	n.a.	n.a.
	4	0.20	416	256	160	n.a.	n.a.	n.a.	n.a.
	5	0.19	317	191	126	n.a.	n.a.	n.a.	n.a.

### Protein preparation and docking

Protein crystal structures were prepared with the protein prep wizard in Maestro 11, Schrödinger Suites 2017-4 [34]. First, modified amino acid residues were mutated back to wild type. Next, the protein was prepared by filling in missing side chains, adding hydrogens, and creation of disulfide bonds. Compounds were prepared for docking using LigPrep from Schrödinger Suites 2017-4. Different tautomers were generated and compound charges were calculated at pH 7.4. Docking was performed with Glide from Maestro 11, Schrödinger Suites 2017-4. SP (standard precision) was used in docking and 10 poses per compound were generated.

## Supplementary information

**Additional file 1.** Compound data used in training and validation (compound smiles and activities).

**Additional file 2.** Most frequent chemical scaffold of compounds that were wrongly predicted by the selectivity-window model.

**Additional file 3.** Protocol to train the selectivity-window model and to apply the model in screening.

**Additional file 4.** KNIME workflow to train the selectivity-window model.

**Additional file 5.** Training dataset including descriptors for model training in KNIME.

## Data Availability

The dataset supporting the conclusions of this article is included within the article and its additional files.

## Abbreviations

A_1_AR: A_1_ adenosine receptor
A_2A_AR: A_2A_ adenosine receptor
cAMP: Cyclic adenosine-5′-monophosphate
FEP: Free-energy perturbation
GPCR: G protein-coupled receptor
MCC: Matthews Correlation Coefficient
NPV: Negative predictive value
PCM: Proteochemometrics
PPV: Positive predictive value
QSAR: Quantitative structure–activity relationship
ROC: Receiver operating characteristic

## References

[1] Burggraaff L, Oranje P, Gouka R (2019). Identification of novel small molecule inhibitors for solute carrier SGLT1 using proteochemometric modeling. J Cheminform.

[2] van Westen GJP, Wegner JK, Geluykens P (2011). Which compound to select in lead optimization? Prospectively validated proteochemometric models guide preclinical development. PLoS ONE.

[3] Lenselink EB, Jespers W, van Vlijmen HWT (2016). Interacting with GPCRs: using interaction fingerprints for virtual screening. J Chem Inf Model.

[4] Lenselink EB, Louvel J, Forti AF (2016). Predicting binding affinities for GPCR ligands using free-energy perturbation. ACS Omega.

[5] Jespers W, Esguerra M, Åqvist J, Gutiérrez-de-Terán H (2019). QligFEP: an automated workflow for small molecule free energy calculations in Q. J Cheminform.

[6] Anighoro A, Bajorath J, Rastelli G (2014). Polypharmacology: challenges and opportunities in drug discovery. J Med Chem.

[7] Jacobson KA, Gao Z-G (2006). Adenosine receptors as therapeutic targets. Nat Rev Drug Discov.

[8] Fredholm BB, IJzerman AP, Jacobson KA (2001). International union of pharmacology. XXV. Nomenclature and classification of adenosine receptors. Pharmacol Rev.

[9] Bernstein FC, Koetzle TF, Williams GJB (1977). The protein data bank: a computer-based archival file for macromolecular structures. J Mol Biol.

[10] Glukhova A, Thal DM, Nguyen AT (2017). Structure of the adenosine A1 receptor reveals the basis for subtype selectivity. Cell.

[11] Cheng RKY, Segala E, Robertson N (2017). Structures of human A1 and A2A adenosine receptors with xanthines reveal determinants of selectivity. Structure.

[12] Draper-Joyce CJ, Khoshouei M, Thal DM (2018). Structure of the adenosine-bound human adenosine A1 receptor–Gi complex. Nature.

[13] Mattedi G, Deflorian F, Mason JS (2019). Understanding ligand binding selectivity in a prototypical GPCR family. J Chem Inf Model.

[14] van Westen GJP, van den Hoven OO, van der Pijl R (2012). Identifying novel adenosine receptor ligands by simultaneous proteochemometric modeling of rat and human bioactivity data. J Med Chem.

[15] Lenselink EB, ten Dijke N, Bongers B (2017). Beyond the hype: deep neural networks outperform established methods using a ChEMBL bioactivity benchmark set. J Cheminform.

[16] Gaulton A, Bellis LJ, Bento AP (2012). ChEMBL: a large-scale bioactivity database for drug discovery. Nucleic Acids Res.

[17] Guo D, Mulder-Krieger T, IJzerman AP, Heitman LH (2012). Functional efficacy of adenosine A2A receptor agonists is positively correlated to their receptor residence time. Br J Pharmacol.

[18] Cappellacci L, Franchetti P, Pasqualini M (2005). Synthesis, biological evaluation, and molecular modeling of ribose-modified adenosine analogues as adenosine receptor agonists. J Med Chem.

[19] Papadatos G, Gaulton A, Hersey A, Overington JP (2015). Activity, assay and target data curation and quality in the ChEMBL database. J Comput Aided Mol Des.

[20] Van Der Maaten L, Hinton G (2008). Visualizing data using t-SNE. J Mach Learn Res.

[21] Rucktooa P, Cheng RKY, Segala E (2018). Towards high throughput GPCR crystallography: in meso soaking of adenosine A2A receptor crystals. Sci Rep.

[22] Kim J, Wess J, van Rhee AM (1995). Site-directed mutagenesis identifies residues involved in ligand recognition in the human A(2a) adenosine receptor. J Biol Chem.

[23] Sciabola S, Stanton RV, Wittkopp S (2008). Predicting kinase selectivity profiles using free-Wilson QSAR analysis. J Chem Inf Model.

[24] Kramer C, Kalliokoski T, Gedeck P, Vulpetti A (2012). The experimental uncertainty of heterogeneous public K_i_ data. J Med Chem.

[25] Subramanian G, Ramsundar B, Pande V, Denny RA (2016). Computational modeling of β-Secretase 1 (BACE-1) inhibitors using ligand based approaches. J Chem Inf Model.

[26] Svetnik V, Wang T, Tong C (2005). Boosting: an ensemble learning tool for compound classification and QSAR modeling. J Chem Inf Model.

[27] Zhao L, Xiang Y, Song J, Zhang Z (2013). A novel two-step QSAR modeling work flow to predict selectivity and activity of HDAC inhibitors. Bioorg Med Chem Lett.

[28] Bento AP (2014). The ChEMBL bioactivity database: an update. Nucleic acids Res.

[29] Frey BJ, Dueck D (2007). Clustering by passing messages between data points. Science.

[30] Dassault Systèmes BIOVIA (2018) BIOVIA Pipeline Pilot

[31] Berthold MR, Cebron N, Dill F (2007). KNIME: the Konstanz information miner. Studies in classification, data analysis, and knowledge organization.

[32] van Westen GJP, Gaulton A, Overington JP (2014). Chemical, target, and bioactive properties of allosteric modulation. PLoS Comput Biol.

[33] Fawcett T (2006). An introduction to ROC analysis. Pattern Recognit Lett.

[34] Schrödinger (2017) Schrödinger Maestro Release 2017-4

