# Supplementary figures and images for: Quantitative prediction of selectivity between the A1 and A2A adenosine receptors
Source: J Cheminform. 2020 May 13;12:33. doi: 10.1186/s13321-020-00438-3 (PMC7222572; doi:10.1186/s13321-020-00438-3)

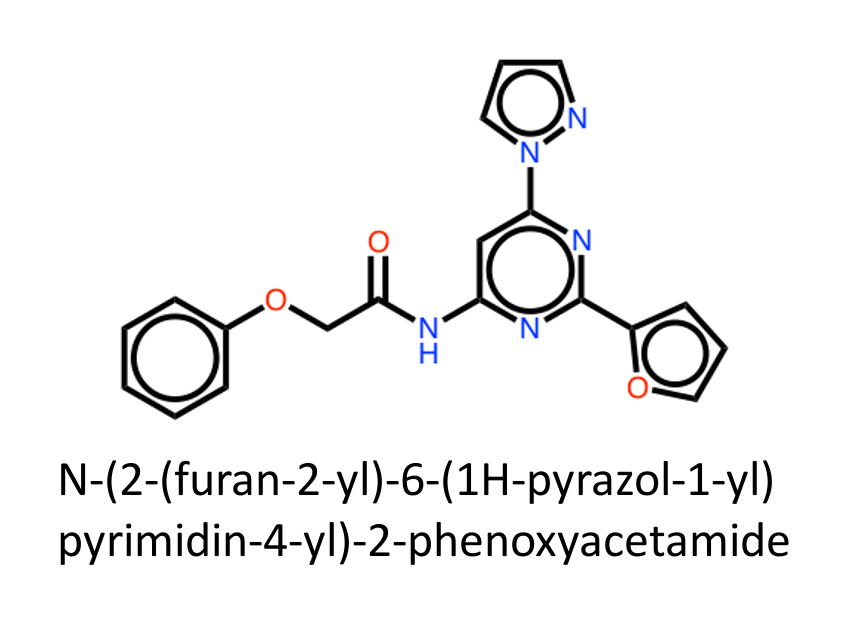

Supplement: Supplementary file 2 — Additional file 2. Most frequent chemical scaffold of compounds that were wrongly predicted by the selectivity-window model. [file 13321_2020_438_MOESM2_ESM.png]
